# Enhanced Wild-Type MET Receptor Levels in Mouse Hepatocytes Attenuates Insulin-Mediated Signaling

**DOI:** 10.3390/cells11050793

**Published:** 2022-02-24

**Authors:** Patricia Rada, Fabienne Lamballe, Elena Carceller-López, Ana B. Hitos, Celia Sequera, Flavio Maina, Ángela M. Valverde

**Affiliations:** 1 Instituto de Investigaciones Biomédicas Alberto Sols, CSIC-UAM, 28029 Madrid, Spain; ecarceller@iib.uam.es (E.C.-L.); ahitos@iib.uam.es (A.B.H.); 2 Centro de Investigación Biomédica en Red de Diabetes y Enfermedades Metabólicas Asociadas (CIBERDEM), 28029 Madrid, Spain; 3 Aix-Marseille Univ, CNRS, Developmental Biology Institute of Marseille (IBDM), Turing Center for Living Systems, Parc Scientifique de Luminy, 13009 Marseille, France; fabienne.lamballe@univ-amu.fr (F.L.); celia.sequera-hurtado@univ-amu.fr (C.S.)

**Keywords:** MET, insulin signaling, hepatocytes, glucose homeostasis

## Abstract

Compelling evidence points to the MET receptor tyrosine kinase as a key player during liver development and regeneration. Recently, a role of MET in the pathophysiology of insulin resistance and obesity is emerging. Herein, we aimed to determine whether MET regulates hepatic insulin sensitivity. To achieve this, mice in which the expression of wild-type MET in hepatocytes is slightly enhanced above endogenous levels (*Alb-R26^Met^* mice) were analyzed to document glucose homeostasis, energy balance, and insulin signaling in hepatocytes. We found that *Alb-R26^Met^* mice exhibited higher body weight and food intake when compared to *R26^stopMet^* control mice. Metabolic analyses revealed that *Alb-R26^Met^* mice presented age-related glucose and pyruvate intolerance in comparison to *R26^stopMet^* controls. Additionally, in *Alb-R26^Met^* mice, high MET levels decreased insulin-induced insulin receptor (IR) and AKT phosphorylation compared to control mice. These results were corroborated in vitro by analyzing IR and AKT phosphorylation in primary mouse hepatocytes from *Alb-R26^Met^* and *R26^stopMet^* mice upon insulin stimulation. Moreover, co-immunoprecipitation assays revealed MET-IR interaction under both basal and insulin stimulation conditions; this effect was enhanced in *Alb-R26^Met^* hepatocytes. Altogether, our results indicate that enhanced MET levels alter hepatic glucose homeostasis, which can be an early event for subsequent liver pathologies.

## 1. Introduction

Receptor tyrosine kinases (RTKs) are high-affinity cell surface receptors for growth factors, hormones, and cytokines. RTKs induce rapid signaling responses at the plasma membrane that are transmitted to intracellular compartments to ultimately modulate the expression of genes relevant to fundamental biological processes including cell proliferation, migration, survival, differentiation, and metabolism [1,2]. MET is a well-known RTK family member widely expressed in many tissues including liver, pancreas, prostate, kidney, and muscle [3,4]. Canonical activation of MET by its cognate ligand hepatocyte growth factor (HGF) triggers a cascade of molecular events including MAPK and AKT signaling pathways, both being common downstream targets of metabolic signaling.

Growing evidence points to a special interest of the HGF/MET axis in the crosstalk with insulin resistance and obesity-related molecular signatures. In particular, HGF has been proposed as an important component of the pathophysiology of insulin resistance-related diseases since both HGF synthesis and secretion are upregulated in insulin resistance conditions (reviewed in [5]). Treatment of obese rats with the combination of recombinant HGF and insulin prevented the decline of insulin receptor (IR) and AKT phosphorylation in the liver. Moreover, other studies have reported that global HGF overexpression ameliorated hepatic steatosis induced by high fat diet (HFD) [6]. Alternatively, the disruption of HGF signaling by daily administration of the MET inhibitor SU11274 during 16 days boosted a reduction in insulin-mediated signaling in diet-induced obese (DIO) rats [7]. Other studies have described that the potentiation of HGF/MET activation by ectopic expression of HGF leads to hepatic, renal, and gut abnormalities [8,9,10,11], whereas MET overexpression confers susceptibility to tumor development [12,13,14]. Therefore, in view of its action in distinct tissues, the impact of tuning HGF/MET modulation on metabolism (and insulin-resistance) should be addressed in a tissue-specific manner.

The action of distinct RTKs should consider how they reciprocally influence downstream signaling and biological outcomes. In this regard, previous reports have illustrated how crosstalks between RTKs lead to the activation of one receptor through signaling pathways mediated by another RTK nearby [15,16]. For example, a bidirectional activation of EGFR and MET in tumor cells has been reported [17,18]. Moreover, the activation of insulin-like growth factor receptor-1 (IGF-1R) delays MET phosphorylation in an HGF-independent manner in the PC3 prostate cancer cell line [19]. Intriguingly, MET is more closely related to the IR than any other member of the RTK family in terms of the overall protein structure and, particularly, of the kinase domain sequence [20]. Regarding molecular metabolic processes, growing evidence suggests that activation of the HGF/MET axis improves glucose metabolism in different insulin sensitive cells such as β-cells, enterocytes, adipocytes, hepatocytes, and myocytes [7,20,21,22,23,24]. This correlates with observations indicating that in hepatocytes, MET directly associates to IR in response to either HGF or insulin, resulting in the recruitment of insulin receptor substrates 1/2 (IRS1/2) and Foxo1 and 4 phosphorylation [20]. MET and IR crosstalk was further supported by in vitro and in vivo experiments using a dominant-negative version of MET (AlbDN-Met), documenting how a functional MET/IR complex is essential to modulate hepatic glucose metabolism by increasing glucose uptake and decreasing hepatic glucose production. Furthermore, hepatocyte-specific deletion of *Met* in mice fed with a methionine-choline deficient diet (MCD) accelerated the development and progression of non-alcoholic steatohepatitis (NASH) [25]. Conversely, *Met* mutant mice exposed to a fast-food diet for 5 months presented similar glucose intolerance and liver steatosis as controls [26]. Nevertheless, the precise role of MET in hepatocytes under insulin resistance conditions is still not fully understood, nor is the impact of subtle changes of MET levels in regular diet regiment. Herein, we genetically assessed how MET inputs impact on hepatic insulin signaling and glucose homeostasis, using conditional transgenic mice in which the expression of wild-type MET is moderately enhanced above endogenous levels (*Alb-R26^Met^* mice) [12]. 

## 2. Materials and Methods

### 2.1. Reagents

Fetal bovine serum (FBS) and culture media were obtained from Thermo Fisher Scientific (Waltham, MA, USA). Protein G-Sepharose was purchased from GE Healthcare (Chicago, IL, USA). Insulin, HGF, and bovine serum albumin (BSA) were from Sigma Aldrich (St. Louis, MO, USA). Bradford reagent, acrylamide, and Clarity™ ECL Western Blotting Substrate were purchased from Bio-Rad Laboratories (Hercules, CA, USA).

### 2.2. Animal Experimentation

The generation of *R26^stopMet^* mice (international nomenclature Gt(ROSA)26Sor^tm1(Actb-Met)Fmai^) carrying a conditional mouse–human chimeric *Met* transgene into the *Rosa26* locus was previously reported [12]. The mouse line expressing the Cre recombinase under the Albumin promoter (B6.Cg-Tg(Alb-cre)21Mgn/J) was obtained from the Jackson Laboratory (Bar Harbor, ME, USA). *Alb-R26^Met^* mice were generated by crossing the *R26^stopMet^* and *Alb-Cre* mice [12,13,14]. Mice were maintained in a 50% mixed background of 129/SV and C57BL6 and genotyped by PCR analysis of genomic DNA, as previously reported [27,28]. Mice were kept in 12 h light/dark cycles, in temperature (22 °C) and humidity-controlled rooms, fed standard chow diet ad libitum, and had free access to drinking water. At 6 and 10 m/o, male mice were used for in vivo studies. For primary hepatocyte isolation, 10 m/o male mice were used. At the end of the in vivo experiments, mice were sacrificed. Blood was collected and processed for biochemical studies. Liver, adipose tissue, and skeletal muscle were snap frozen and stored at −80 °C for subsequent molecular analyses. All animal experimentations were carried out in accordance with the recommendations of the Federation of European Laboratory Animal Science Associations (FELASA) on health surveillance, the European Community Law (2010/63/UE), the Spanish Law (RD 53/2013) with the approval of the Ethics Committee of the Bioethics Commission of the CSIC (Spain), and the French law (under an agreement number E1305521, Ministère de l’Enseignement Supérieur de la Recherche et de l’Innovation) with the institutional Ethical Committee guidelines for animal research (comité d’éthique pour l’expérimentation animale—Comité d’éthique de Marseille).

### 2.3. Transcriptome Analysis by RNA-Seq

Liver samples from *Alb-R26^Met^* and *R26^stopMet^* mice were submitted to GATC Biotech for RNA sequencing with Illumina paired-end technology (*n* = 4 *R26^stopMet^* and 3 *Alb-R26^Met^*) following the methodology previously reported [12]. The differential expression of genes was calculated as log2FC, and their significance was assessed.

### 2.4. Analysis of Publicly Available RNA-Seq Data

Data from other mouse models and human samples were downloaded and extracted from available public datasets. We used GSE182668 from NCBI GEO Database in which RNA-seq was performed in livers from male mice of nine different strains fed a chow diet (CD) or HFD. Furthermore, RNA-seq data were extracted from GSE10991 from NCBI GEO database, and the data correspond to a cohort of 910 obese patients with different degrees of type 2 diabetes (T2D). In this case, patients have been separated by sex.

### 2.5. Analysis of Plasma Insulin Levels

Plasma was collected in fed and 16-hour-fasted conditions from blood obtained from the tail vein. In particular, blood was centrifuged 20 min at 11,600× *g* at 4 °C; plasma was then collected and snap frozen. Plasma insulin levels were measured by ELISA in accordance with manufacturing guidelines (Mercodia, Uppsala, Sweden). Briefly, 10 µL of the samples was placed per well in a 96-well plate as part of the ELISA kit and 100 µL of the conjugated enzyme was added to each well. The plate was incubated protected from light under rotation for 2 h at RT, washed 5 times to remove unbound enzyme labelled antibody, and then dried. The bound conjugate was detected by reaction with 200 µL of 3,3′,5,5′-tetramethylbenzidine (TMB). The reaction was stopped by adding 50 µL of stop solution (hydrochloric acid based) to give a colorimetric endpoint that was read spectrophotometrically at 450 nm. 

### 2.6. Glucose, Insulin, and Pyruvate Tolerance Tests (GTT, ITT, PTT)

For glucose tolerance tests, 16 h fasted mice received an intraperitoneal (i.p.) injection of 2 g D-glucose/kg body weight. Glucose concentration in blood was measured with an Accu-Check glucometer (Roche Diagnostics, Basel, Switzerland) at 0, 15, 30, 60, 90, and 120 min time points, and the area under the curve (AUC) was calculated. For insulin tolerance tests, 4 h fasted mice received an i.p. injection of 0.75 U insulin/kg body weight. Pyruvate tolerance tests were performed similarly to GTT, with the exception that the mice received an i.p. injection of 1.5 g sodium pyruvate/kg body weight. 

### 2.7. Indirect Calorimetry

Indirect calorimetry analyses were carried out using a 16-chamber TSE PhenoMaster monitoring system (TSE Systems GmbH, Bad Homburg, Germany). *Alb-R26^Met^* and *R26^stopMet^* mice were placed in acclimatization cages similar to experimental cages, with one mouse per cage. Acclimation starts the Friday before the experiment, with the experiment running for 5 consecutive days. Mice were on a 12 h light–dark cycle, and room temperature was maintained at 22 ± 2 °C. Food and water were provided ad libitum in appropriated devices that allow to measure cumulative food intake and drink consumption. The determination of different parameters was carried out over a period of 84 h. The respiratory exchange ratio (RER) was estimated by calculating the ratio of VCO_2_/VO_2_. Energy expenditure (H(1)) was calculated as follows: H(1) = (3.185 + 1.232 × RER) × VO_2_. Total locomotor activity (ambulatory and fine) was measured simultaneously along the x and y axes using an infrared photocell beam grid and represented as the average of the total number of beam breaks along the x and y axes during light and dark periods.

### 2.8. Homogenization and Preparation of Tissue Extracts

Frozen liver and eWAT tissues were homogenized in ice-cold lysis buffer containing 50 mM Tris-HCl, 1% Triton X-100, 2 mM EGTA, 10 mM EDTA acid, 100 mM NaF, 1 mM Na_4_P_2_O_7_, 2 mM Na_3_VO_4_, 100 µg/mL phenylmethylsulphonyl fluoride (PMSF), and 2.5 μg/mL protease inhibitors by using the Brinkman PT 10/35 Polytron. Extracts were always kept ice-cold. Tissue extracts were cleared twice by ultracentrifugation at 40,000× *g* for 40 min at 4 °C. Protein concentration was determined by using the Pierce BCA Protein Assay Kit (Thermo Fisher Scientific, Waltham, MA, USA). 

Muscle extracts were prepared in cold lysis buffer containing 50 mM Tris-HCl pH 7.5, 1 mM EGTA, 1 mM EDTA, 50 mM NaF, 1 mM sodium β-glycerophosphate, 5 mM sodium pyrophosphate, 0.27 M sucrose, 1% Triton X-100, 0.1% β-mercaptoethanol, 1 mM Na_3_VO_4,_ 100 µg/mL PMSF, and 2.5 μg/mL protease inhibitors by using te Brinkman PT 10/35 Polytron. Lysates were centrifuged at 19,000× *g* for 20 min at 4 °C, and the protein content of the supernatants was determined by the Bradford method (Bio-Rad Laboratories, Hercules, CA, USA).

### 2.9. Isolation and Culture of Primary Mouse Hepatocytes

Primary mouse hepatocytes were isolated from non-fasting male mice by using a two-step collagenase perfusion as previously described [29]. Cells were seeded on 6 or 12-well collagen IV pre-coated plates, cultured in media containing DMEM and Ham’s F-12 medium (1:1) with heat-inactivated 10% FBS, supplemented with 2 mm glutamine, 15 mM glucose, 20 mM HEPES, 100 U/mL penicillin, 100 µg/mL streptomycin, and 1 mm sodium pyruvate (attachment media) and maintained in this medium for 24 h. Then, the medium was changed to DMEM 5.5 mM glucose 2 h prior to HGF (20 and 40 ng/mL, 15 min) or insulin (10 nM, 5 and 15 min) stimulation. 

### 2.10. Western Blot Analysis

After culture and treatments, cells were washed twice with PBS, then scraped off in lysis buffer containing 10 mM Tris-HCl, 5 mM EDTA, 50 mM NaCl, 30 mM disodium pyrophosphate, 50 mM NaF, 100 µM Na_3_VO_4_, 1% Triton X-100, 100 µg/mL PMSF, and 2.5 μg/mL protease inhibitors (pH 7.6). Cellular lysates were clarified by centrifugation at 12,000× *g* for 10 min. Protein content was determined by the Bradford method (Bio-Rad Laboratories, Hercules, CA, USA). Protein extracts were boiled at 95 °C for 5 min in a loading buffer (100 mM Tris, pH 6.8, 10% glycerol, 4% sodium dodecyl sulphate (SDS), 0.2% bromophenol blue, and 2 mM β-mercaptoethanol), subjected to SDS polyacrylamide gel electrophoresis (SDS-PAGE), and then transferred to PVDF membranes (Immobilon-P IPVH00010, Merck, Darmstadt, Germany). Membranes were blocked with 5% non-fat dry milk or 4% BSA in TBS supplemented with 0.05% Tween-20 (TTBS) for 2 h at RT and then incubated with primary antibodies, as indicated in TTBS, overnight at 4 °C. After 3 washes with TTBS, membranes were incubated with the corresponding peroxidase-conjugated secondary antibodies (anti-rabbit, 1/15,000, A120-108P, Bethyl Laboratories; anti-mouse, 1/10,000, sc-2005, Santa Cruz Biotechnologies, Dallas, TX, USA) for 1 h at RT. Membranes were developed with chemiluminescent substrate (Clarity Western ECL Substrate, 170-5060, Bio-Rad Laboratories, Hercules, CA, USA) and different exposure times were performed for each primary antibody with radiographic films in a radiology cassette (AGFA) or developed in a ChemiDoc imager (Bio-Rad Laboratories, Hercules, CA, USA). Primary antibodies used are listed in Table 1. Blots were normalized using antibodies against housekeeping proteins. Densitometry values were determined using Image J. 

### 2.11. Co-Immunoprecipitation Assays

Cells were scraped off in lysis buffer containing 10 mM Tris-HCl, 5 mM EDTA, 50 mM NaCl, 30 mM disodium pyrophosphate, 50 mM NaF, 100 µM Na_3_VO_4_, 1% Triton X-100, 1 mM PMSF, and 2.5 μg/mL protease inhibitors (pH 7.6). Cellular lysates were clarified by centrifugation at 12,000× *g* for 10 min. Protein content was determined by the Bradford method (Bio-Rad), and equal amounts of proteins (500 µg) were immunoprecipitated at 4 °C with the anti-IR antibody (sc-57342, Santa Cruz Biotechnologies, Dallas, TX, USA). Immune complexes were collected on G-Sepharose beads and subjected to Western blot analysis.

### 2.12. Data Analysis

Statistical analysis was performed using the GraphPad Prism software version-8.0. Data are reported as mean and standard error of the mean (SEM). Normal distribution was assessed by two different tests: Shapiro–Wilk and Kolmogorov–Smirnov tests. Statistical details are indicated in the figure legends. Differences were considered statistically significant at *p* < 0.05. For correlations, Pearson coefficient was calculated.

## 3. Results

### 3.1. A Slight Increase in MET Levels Perturbs the Expression of a Set of Genes Related to Insulin Signaling

**Figure 1:**
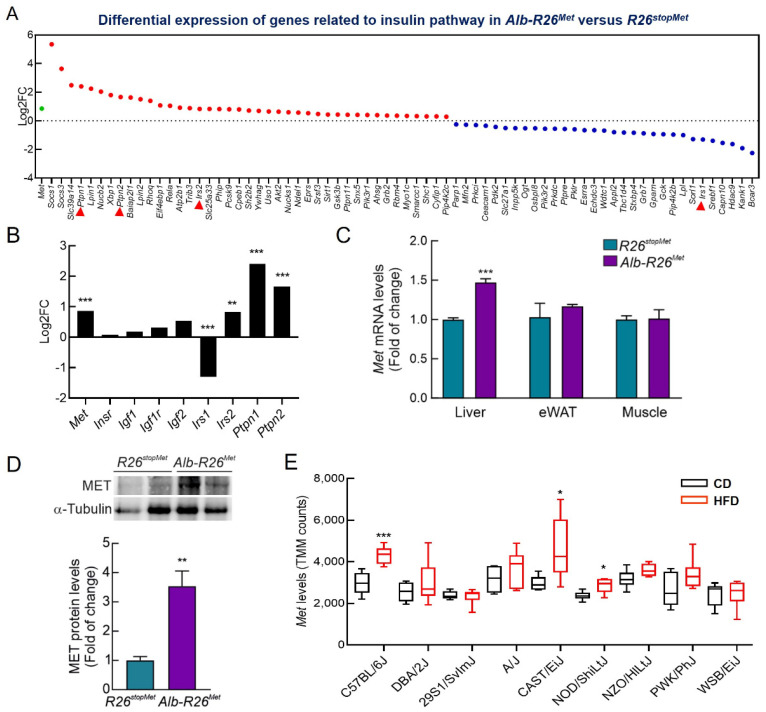
A slight increase in *Met* levels perturbs the expression of a set of genes related to insulin signaling. (**A**) Dotplot reporting the genes related to insulin signaling pathway that are upregulated (red) or downregulated (blue) resulting from differential expression (DE) analysis and calculated as log2FC of *Alb-R26^Met^* mutant (3 replicates) versus *R26^stopMet^* control livers (4 replicates) analyzed by RNA-seq. Only genes with significant DE are represented (*p* < 0.05). *Met* expression is also included (green). (**B**) Histogram showing log2FC of *Met*, different ligands (*Igf1* and *Igf2*), mediators (*Ptpn1*, *Ptpn2*, *Irs1*, and *Irs2*) and receptors (*Insr* and *Igf1r*) related to the insulin pathway in *Alb-R26^Met^* mutant versus *R26^stopMet^* control livers. (**C**) *Met* mRNA levels determined by qRT-PCR in liver, epididymal white adipose tissue (eWAT), and skeletal muscle from in *R26^stopMet^* and *Alb-R26^Met^* males (*n* = 3 and 6 mice per group, respectively). *Tbp* mRNA levels were used as endogenous control. (**D**) MET protein levels determined by Western blot in liver in *R26^stopMet^* and *Alb-R26^Met^* mice (*n* = 4 and 3 mice per group, respectively). α-Tubulin was used as loading control. Densitometric quantification of MET protein levels. (**E**) Boxplot reporting *Met* levels, expressed as TMM counts, resulting from the RNA-seq analysis deposited in the available GSE182668 GEO database. Male mice from 9 different strains were fed with either chow diet (CD, black) or high fat diet (HFD, red), *n* = 6 mice per group. Statistical analysis was performed according to Student’s *t* test (* *p* < 0.05, ** *p* < 0.01, *** *p* < 0.001).

We have previously reported the generation of the *Rosa26^LacZ-stop-Met^* mice (herein referred as *R26^stopMet^*), which allowed wild-type MET levels to increase in a temporal and spatial-regulated manner [12]. To specifically enhance MET in hepatocytes, *Alb-R26^Met^* mice were generated by crossing *R26^stopMet^* and *Alb-Cre* mice [12]. This genetic setting allows exploring the impact of hepatocyte-specific modest MET upregulation in whole-body glucose homeostasis. Using RNA-seq data from *Alb-R26^Met^* mutant and *R26^stopMet^* control livers, we found changes in several genes implicated in the insulin signaling pathway (Go_term = 133,216; Figure 1A). Interestingly, genes acting as negative regulators increased, such as *Ptpn1* and *Ptpn2* (encoding PTP1B and TCPTP, respectively) [30,31] (Figure 1A,B). Additionally, we found a downregulation of *Irs1* accompanied by an upregulation of *Irs2.* In contrast, no significant changes were observed for insulin pathway-related ligands (*Igf1* and *Igf2*) or receptors (*Insr* and *Igf1r*; Figure 1A,B). As shown in Figure 1C, enhanced hepatic *Met* expression was validated in *Alb-R26^Met^* mice when compared to *R26^stopMet^* control mice by determining *Met* mRNA levels by RT-qPCR, as previously reported [12]. We also measured *Met* mRNA levels in epididymal white adipose tissue (eWAT) and skeletal muscle and, reassuringly no differences were found genotypes. In addition, the analysis of MET protein levels in the liver showed that the *Alb-R26^Met^* mice presented an approximately 3.5-fold increase compared to *R26^stopMet^* mice (Figure 1D), as previously shown [12]. Hence, a slight increase in MET levels in *Alb-R26^Met^* livers deregulates components of the insulin signaling pathways that, among other metabolic insulin actions, regulate glucose homeostasis.

To obtain further insights on how *Met* levels may be linked to metabolic-associated perturbations related to insulin action, we assessed whether *Met* levels are influenced by a type of diet that challenges insulin signaling and glucose homeostasis, using RNA-seq data from other mouse models (GEO Database with accession number GSE182668). We compared *Met* levels in mice fed a high fat diet (HFD), leading to obesity, versus a normal chow diet (CD). Results showed a trend toward increased *Met* levels, which was significant in three mouse strains (Figure 1E). Collectively, these results suggest that a slight increase in *Met* levels may be associated with insulin signaling modulation and metabolic processes related to glucose homeostasis.

### 3.2. A Slight Enhancement of MET Levels in the Liver Results in Increased in Body Weight and Food Intake

**Figure 2:**
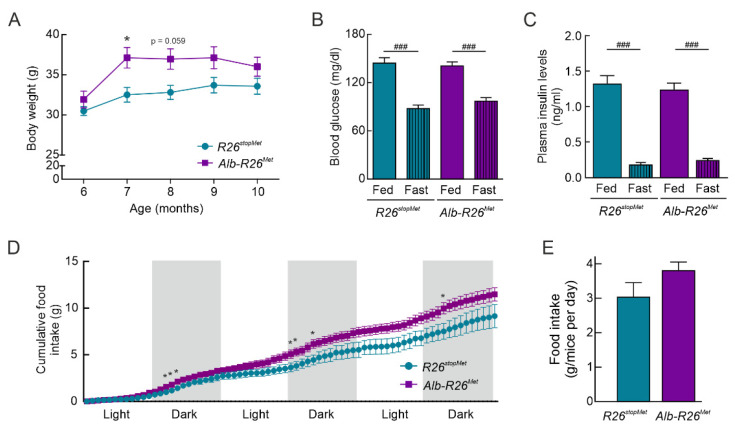
*Alb-R26^Met^* mice present elevated body weight and food intake compared to *R26^stopMet^* controls. (**A**) Body weight evolution and total body weight gain of *R26^stopMet^* and *Alb-R26^Met^* mice (*n* = 10 and 19 mice per group, respectively). (**B**) Blood glucose levels (mg/dl) under fed and 16 h fasting conditions in *R26^stopMet^* and *Alb-R26^Met^* mice (*n* = 9 and 19 mice per group, respectively). (**C**) Plasma insulin levels (ng/mL) in fed (*n* = 4 and 14 mice per group, respectively) and 16 h fasted (*n* = 6 and 8 mice per group, respectively) conditions. (**D**) Cumulative food intake (g) of *R26^stopMet^* and *Alb-R26^Met^* mice (*n* = 6 and 10 mice per group, respectively). (**E**) Food intake (g) (*n* = 6 and 10 mice per group, respectively). Blood glucose, plasma insulin, and food intake were measured at the end of the experiment and, therefore, in 10 months old mice. (**A**–**E**) Values are mean ± SEM. For (**A**) and (**D**), statistical analysis was performed by fitting a mixed model followed by a Bonferroni post hoc test. For (**B**), statistical analysis was performed by a two-way ANOVA followed by a Bonferroni post hoc test. For (**C**) and (**E**), statistical analysis was performed according to a Student’s *t* test. * *p* < 0.05, ** *p* < 0.01, *** *p*< 0.001vs. *R26^stopMet^* mice. ^###^ *p* < 0.001 vs. Fed condition.

We observed that *Alb-R26^Met^* mice showed a slight increase in both body weight evolution and total body weight gain compared to their *R26^stopMet^* littermates (Figure 2A), although no significant differences were obtained at the end of the experiment when mice aged 10 months. However, no differences were detected in blood glucose (Figure 2B) and plasma insulin levels (Figure 2C) neither in fed nor fasting conditions analyzed at the end of the experiment. Moreover, analysis of food intake at the same time period revealed an increased trend in *Alb-R26^Met^* compared to *R26^stopMet^* mice; this effect was statistically significant during the first 4–5 h of the dark phase (Figure 2D,E).

### 3.3. Hepatocyte-Specific Enhanced MET Levels Impair Glucose Homeostasis In Vivo

**Figure 3:**
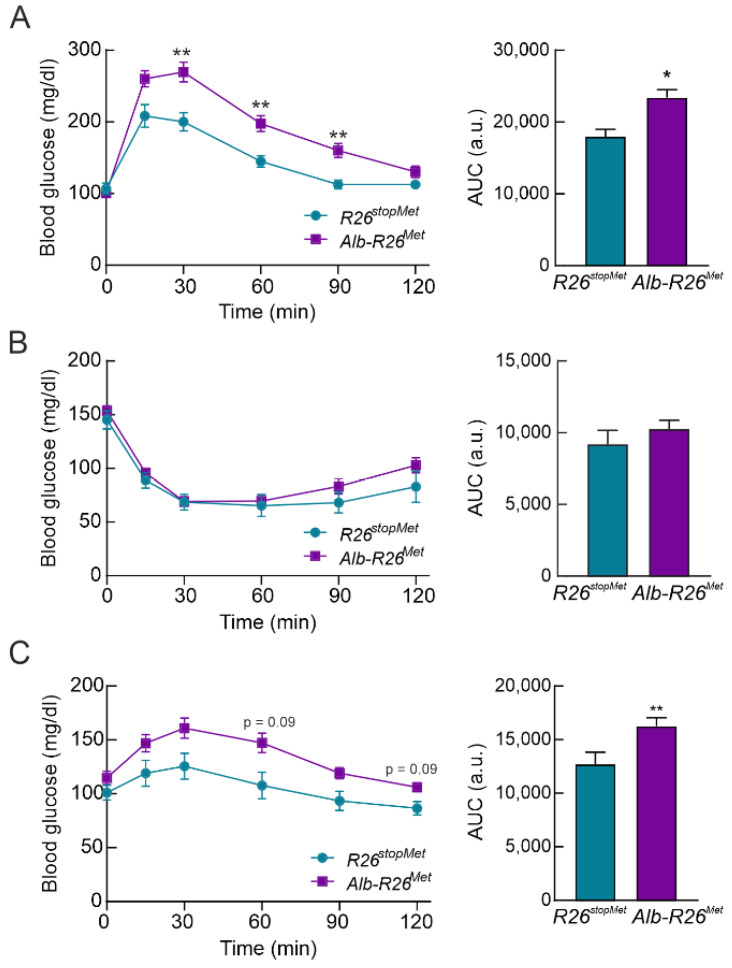
Enhanced MET levels in hepatocytes impairs glucose homeostasis. (**A**) Glucose tolerance test (GTT) in *R26^stopMet^* and *Alb-R26^Met^* mice (*n* = 11 and 38 mice per group, respectively). Graph depicts the area under the curve (AUC) from GTT. (**B**) Insulin tolerance test (ITT) in *R26^stopMet^* and *Alb-R26^Met^* mice (*n* = 10 and 39 mice per group, respectively). Graph depicts AUC from ITT. (**C**) Pyruvate tolerance test (PTT) in *R26^stopMet^* and *Alb-R26^Met^* mice (*n* = 14 and 35 mice per group, respectively). Graph depicts AUC from PTT. (**A**–**C**) Values correspond to mean ± SEM. For time-course studies, statistical analysis was performed by a two-way repeated measures (RM) ANOVA followed by a Bonferroni post hoc test. For AUC, statistical analysis was performed according a Student’s *t* test. * *p* < 0.05, ** *p* < 0.01 vs. *R26^stopMet^* mice.

Results from the glucose tolerance test (GTT) revealed that increased MET levels in hepatocytes led to a significant glucose intolerance (Figure 3A). However, insulin sensitivity was similar in both groups according to the insulin tolerance test (ITT) (Figure 3B). Since approximately 85% of circulating glucose is disposed by skeletal muscle, a pyruvate tolerance test (PTT) was performed to analyze exclusively the response of the liver, where MET is increased. As shown in Figure 3C, PTT revealed a significant pyruvate intolerance in *Alb-R26^Met^* compared to *R26^stopMet^* mice. In addition, 6-month-old *Alb-R26^Met^* mice had mild pyruvate intolerance, showing that impaired glucose homeostasis worsens with age (Figure S1). Altogether, these results raise the possibility that hepatic MET levels may be involved in the control of hepatic glucose output.

### 3.4. Enhanced MET Levels in Hepatocytes Do Not Severely Alter Whole-Body Energy Metabolism

**Figure 4:**
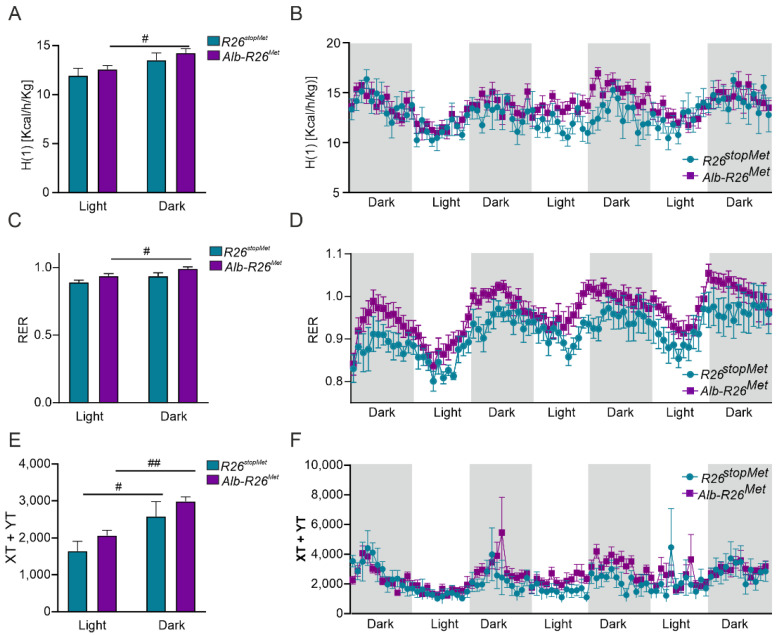
Effect of hepatocyte-specific enhanced MET levels in whole-body energy balance. (**A**) Energy expenditure (H(1) [Kcal/h/Kg]). (**B**) H(1) vs. time in *R26^stopMet^* and *Alb-R26^Met^* mice. (**C**) Respiratory Exchange Ratio (RER) in *R26^stopMet^* and *Alb-R26^Met^* mice. (**D**) RER vs. time in *R26^stopMet^* and *Alb-R26^Met^* mice. (**E**) Spontaneous locomotor activity (XT + YT) in *R26^stopMet^* and *Alb-R26^Met^* mice. (**F**) XT + YT vs. time in *R26^stopMet^* and *Alb-R26^Met^* mice. (**A**–**F**) Values correspond to mean ± SEM (*n* = 6 and 10 mice per group, respectively). For a, c, and e, statistical analysis was performed by a two-way ANOVA followed by a Bonferroni post hoc test. ^#^ *p* < 0.05, ^##^ *p* < 0.01 vs. light phase.

To further assess whether hepatic-specific increased MET levels had an impact on whole-body energy homeostasis, we measured the metabolic activity of the mice by using indirect calorimetry (TSE Systems PhenoMaster). We found that *Alb-R26^Met^* mice exhibited a significant increase in energy expenditure in the dark phase compared to the light phase (Figure 4A,B). However, no changes among phases were detected in the *R26^stopMet^* control group. In addition, slight increases in the respiratory exchange ratio (RER) were observed in *Alb-R26^Met^* mice in both light and dark cycles (Figure 4C,D). These differences in RER, although not statistically significant, suggest that *Alb-R26^Met^* mice exhibited a preference for carbohydrate oxidation reflected by higher RER values compared to *R26^stopMet^* controls. No substantial differences in spontaneous locomotor activity between genotypes were observed, although *Alb-R26^Met^* mice reached higher values in both dark and light phases (Figure 4E,F). Overall, these results indicate that, despite some specific changes, enhanced MET levels in hepatocytes do not drastically impact on whole-body energy homeostasis.

### 3.5. Enhanced MET Levels Restrain Insulin Signaling In Vivo

**Figure 5:**
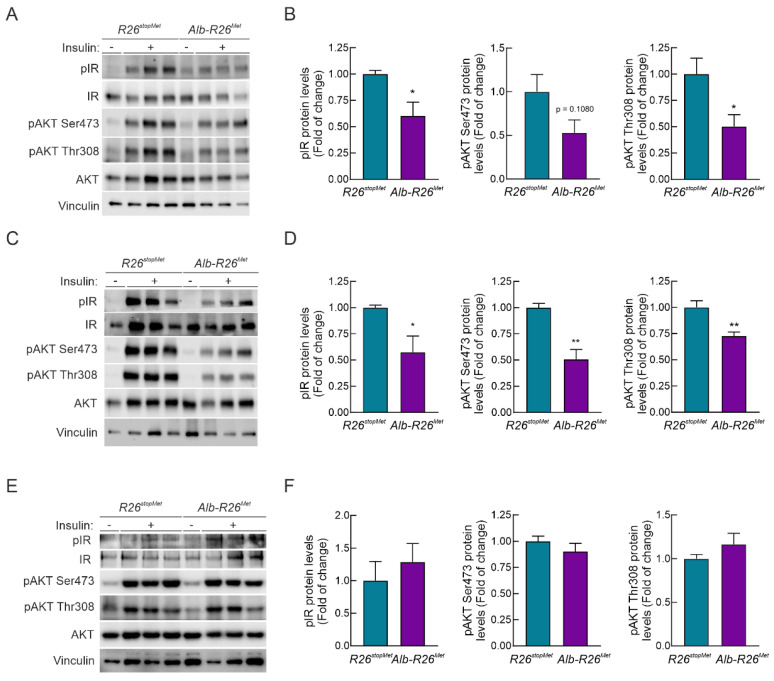
Hepatocyte enhanced MET levels lead to impaired insulin sensitivity in liver and in eWAT, but not in skeletal muscle. *R26^stopMet^* and *Alb-R26^Met^* mice were fasted 4 h and then submitted to insulin stimulation (i.p. injection, 0.75 U/Kg) for 15 min prior to sacrifice. Liver, epididymal white adipose tissue (eWAT) and skeletal muscle were collected. (**A**) Phosphorylation levels of IR and AKT (pIR and pAKT) in liver extracts from *R26^stopMet^* and *Alb-R26^Met^* mice (*n* = 6 and 9 mice per group, respectively). IR, AKT, and Vinculin were used as loading controls. (**B**) Densitometric quantification of the indicated protein levels shown in (**A**). (**C**) Phosphorylation levels of IR and AKT (pIR and pAKT) in eWAT extracts from *R26^stopMet^* and *Alb-R26^Met^* mice (*n* = 3 and 5 mice per group, respectively). IR, AKT, and Vinculin were used as loading controls. (**D**) Densitometric quantification of the indicated protein levels shown in (**C**). (**E**) IR (*n* = 3 mice per group) and AKT (*n* = 6 and 10 mice per group, respectively) phosphorylation levels in skeletal muscle extracts from *R26^stopMet^* and *Alb-R26^Met^* mice. AKT, IR, and Vinculin were used as loading controls. (**F**) Densitometric quantification of the indicated protein levels shown in (**E**); (**B**,**D**,**F**) Values are mean ± SEM. Statistical analysis was performed according to Student’s *t* test. * *p* < 0.05, ** *p* < 0.01 vs. *R26^stopMet^* mice.

We next explored in vivo how enhanced MET levels impact the response of hepatocytes to insulin stimulation. *Alb-R26^Met^* and *R26^stopMet^* mice were fasted for 4 h and then i.p. injected with insulin (0.75 U/kg) for 15 min. Liver, epididymal white adipose tissue (eWAT), and skeletal muscle were then collected for biochemical studies. Strikingly, phosphorylation of IR and AKT were significantly reduced in *Alb-R26^Met^* livers compared to *R26^stopMet^* controls (Figure 5A,B). These results indicate that hepatocyte enhanced MET levels promote a molecular signature of insulin resistance in the liver. Intriguingly, we found a reduction in insulin signaling in eWAT extracts from *Alb-R26^Met^* mice compared to controls (Figure 5C,D). These results are consistent with a gain of weight in *Alb-R26^Met^* compared to control mice (Figure 2A). In contrast, insulin-induced IR and AKT phosphorylation in skeletal muscles was comparable in both genetic settings (Figure 5E,F), coherent with ITT results (Figure 3B). Collectively, these data show that the impacts of MET levels on hepatic glucose homeostasis correlate with insulin signaling modulation.

### 3.6. Enhanced MET Levels Attenuate Insulin Sensitivity in Primary Adult Hepatocytes

**Figure 6:**
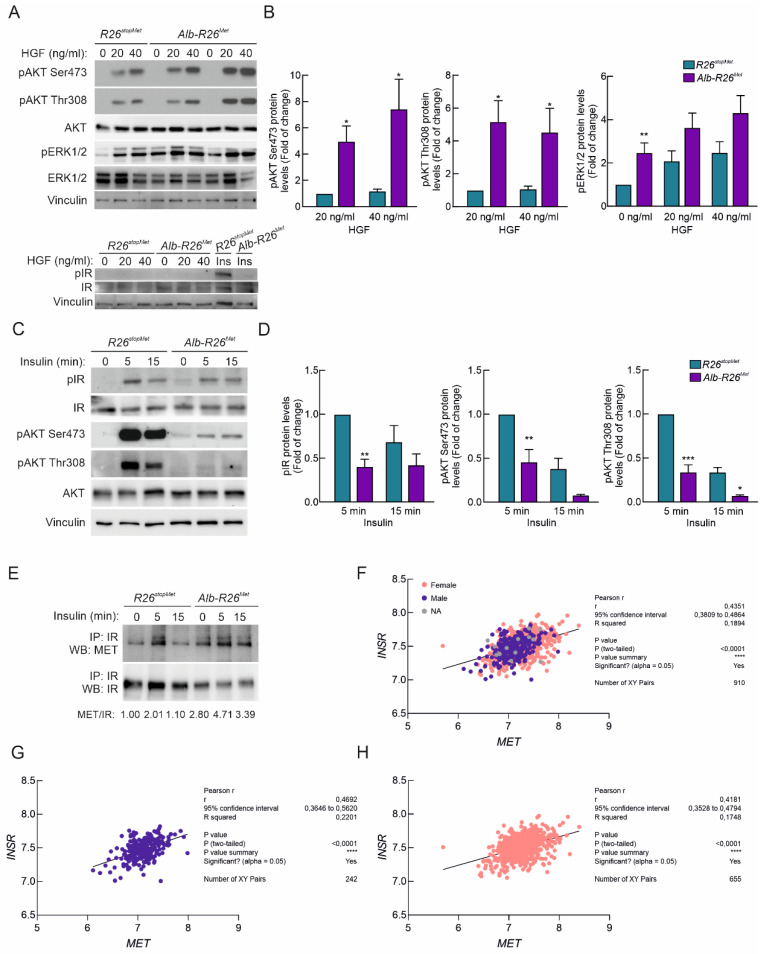
Hepatic enhanced MET levels lead to reduced insulin sensitivity in primary hepatocytes. (**A**) Primary mouse hepatocytes from *R26^stopMet^* and *Alb-R26^Met^* mice were exposed to HGF (40 ng/mL) for 15 min. Upper panel: phosphorylation levels of pAKT and pERK1/2 were analyzed. Vinculin, AKT, and ERK1/2 were used as loading controls. Lower panel: phosphorylation levels of IR were analyzed. IR and Vinculin were used as loading controls. (**B**) Densitometric quantification of the indicated protein levels shown in (**A**). (**C**) Primary mouse hepatocytes from *R26^stopMet^* and *Alb-R26^Met^* mice were exposed to insulin (10 nM) for 5–15 min. Phosphorylation levels of IR and AKT were analyzed. IR, AKT, and Vinculin were used as loading controls. (**D**) Densitometric quantification of the indicated protein levels shown in (**C**). (**E**) IR was immunoprecipitated from *R26^stopMet^* and *Alb-R26^Met^* primary hepatocytes and IR and MET protein levels were analyzed by Western blot. Experiments were performed in primary hepatocytes from at least 3 independent mice per group. (**B**–**D**) Values are mean ±SEM. Statistical analysis was performed according to Student’s *t* test and two-way ANOVA followed by a Bonferroni post hoc test. * *p* < 0.05, ** *p* < 0.01, *** *p*< 0.001 vs. R26^stopMet^ mice. (**F**–**H**) Graph reporting *MET* and *INSR* expression levels for obese patients separated by sex (blue: men; pink: women) with different grades of T2D (*n* = 910), resulting in significant positive correlation as indicated by Pearson’s analysis. Data were extracted and analyzed from the RNA-seq deposited in the GSE10991 GEO database. Statistical analysis was performed according to Pearson’s coefficient (*p* < 0.0001).

Next, we assessed the impact of slightly enhanced MET levels on the perception of HGF and insulin stimulation in primary adult hepatocytes isolated from *Alb-R26^Met^* and *R26^stopMet^* mice. While no basal AKT phosphorylation was observed in *Alb-R26^Met^* and *R26^stopMet^* primary hepatocytes, an increase in ERK1/2 phosphorylation was found in *Alb-R26^Met^* cells (Figure 6A,B). Notably, HGF stimulation (20 and 40 ng/mL; for 15 min) promoted both AKT and ERK1/2 phosphorylation, and levels were more pronounced in *Alb-R26^Met^* hepatocytes compared to controls (Figure 6A,B). However, HGF did not trigger IR phosphorylation in hepatocytes from both genotypes (Figure 6A), suggesting the inability of HGF to activate the insulin signaling cascade. These results show that increased MET levels in adult mouse hepatocytes are accompanied by enhanced signaling in response to HGF.

Next, we tested the cell autonomous effect of increased MET levels on insulin sensitivity. Primary *Alb-R26^Met^* and *R26^stopMet^* mouse hepatocytes were serum-starved in DMEM (5.5 mM glucose) 2 h prior to insulin stimulation (10 nM, 5–15 min). Notably, we found that insulin-mediated IR phosphorylation was reduced in *Alb-R26^Met^* primary adult hepatocytes compared to controls (Figure 5C,D). This was accompanied by a drastic reduction in AKT phosphorylation on both Ser473 and Thr308 (Figure 6C,D). These results are consistent with reduced IR and AKT phosphorylation observed in *Alb-R26^Met^* livers (Figure 5). These findings document that enhanced MET levels lead to reduced hepatic insulin sensitivity. Next, we analyzed a putative interaction between MET and IR and assessed how this interaction is influenced by enhanced MET levels in adult hepatocytes (Figure 6E). Interestingly, co-immunoprecipitation studies revealed MET-IR interaction under basal conditions in *Alb-R26^Met^* hepatocytes, which was potentiated and sustained following insulin stimulation. These findings are consistent with a previous report showing that MET activation in HGF-treated hepatocytes induced the formation of a MET-IR complex [20]. Moreover, positive correlations between *MET* and *INSR* levels were found in a cohort of obese patients separated by sex, with different grades of T2D (Figure 6F–H).

## 4. Discussion

The HGF/MET system is essential during liver development [32,33,34,35,36,37] and has a fundamental role in regeneration and hepatoprotection against liver injury [38,39,40]. MET deregulation is frequent in patients with hepatocellular carcinoma (HCC) and plays a key role in the tumorigenic properties of HCC cells [12]. In cancer, the interplay between MET and other RTKs, either through RTK coactivation or RTK switching, has profound implications in the mechanisms of resistance to anticancer chemotherapy [41,42,43]. Among several examples, a recent study showed that an EGFR/MET heterodimer interacts with and phosphorylates PARP1 at tyrosine 907 in the nucleus, thus contributing to PARP inhibition resistance [44]. In this line, combined EGFR/MET inhibition sensitized HCC cells to these chemotherapeutic agents [45].

In the context of metabolic diseases, the HGF/MET system has uncertain mixed functions. While HGF/MET can improve the metabolic profile of T2D, it conversely induces insulin resistance [5,46,47]. Regarding the latter, a significant association between HGF and clinical/anthropometric features of metabolic syndrome has been found in the study of Hiratsuka and coworkers [48]. The association between insulin resistance and HGF levels was supported by two additional prospective studies [49,50], suggesting that insulin and HGF resistance might converge. However, at the molecular level, the interplay between the signaling cascades activated by their reciprocal IR and MET RTKs, which are structurally related [5,20], remained elusive. In the present study, we used the unique genetic setting of *Alb-R26^Met^* mice [27] to examine how a moderate increase in MET levels in hepatocytes impact whole-body glucose homeostasis and energy balance, as well as hepatic insulin responses. To the best of our knowledge, this approach is novel in the context of mediators of hepatocyte insulin sensitivity.

RNA-seq data analysis from the *Alb-R26^Met^* mutant and *R26^stopMet^* control livers revealed notable changes in the expression of genes related to the insulin signaling pathway such as *Ptpn1* and *Ptpn2*, which are both negative modulators of insulin signaling [30,31] concurring with a downregulation of *Irs1.* Thus, augmented MET levels in the liver are sufficient for deregulating insulin signaling-related gene signature. Taking a step further, the comparative analysis between *Alb-R26^Met^* mutant and *R26^stopMet^* control mice revealed an increase in body weight, an effect that could be due to a trend of mutants to hyperphagia. Of relevance, in mice with diet-induced obesity, a trend towards an increase in *Met* levels was found in several mouse strains; an effect being significantly different in three of them. On that basis, the present study has been conducted in mice from 6 to 10 months of age fed a normal chow diet to avoid possible masking effects derived from additional metabolic stressors (i.e., HFD).

Indirect calorimetry revealed a predominant use of carbohydrates as substrate fuel in *Alb-R26^Met^* mice, an effect likely associated with an increase in adiposity concomitantly to weight gain. Regarding glucose homeostasis, *Alb-R26^Met^* mice displayed aging-related glucose and pyruvate intolerance. It is intriguing to observe that our MET gain-of-function approach documents glucose intolerance and hepatic insulin resistance, which have been previously reported in a mouse model conceived to work as a MET loss-of-function, based on the expression of an extracellular portion of MET in the liver (AlbDN-Met) [20,51]. Intriguingly, a similar apparent contradiction has been reported in a different study exploring the in vivo effects of the Fas/CD95 death receptor on hepatocytes. Specifically, it has been shown that Fas/CD95 induced a massive hepatocyte death in loss-of-function liver-specific *Met* mutant mice [52], while loss-of-function AlbDN-Met was resistant to Fas/CD95-induced death [51]. These two apparent contradictions might be linked to some specific features of the AlbDN-Met model, in which the mode of actions still needs to be fully understood.

Another relevant set of data we documented in the present study is related to insulin signaling defects in *Alb-R26^Met^* livers, which strikingly correlated with hepatocyte-specific insulin resistance. These defects are consistently illustrated by reduced insulin responses in IR and AKT phosphorylation, two critical signaling mediators of the metabolic actions of insulin in hepatocytes [53]. Decreased insulin signaling was also found in eWAT, but not skeletal muscle from *Alb-R26^Met^* mice. A plausible explanation for this specific effect in eWAT could rely on potential higher sensitivity of this fat depot to the liver-derived secretome (i.e., hepatokines, extracellular vesicles, as reviewed [54,55]) due to enhanced MET levels specifically in the liver. Attenuation of insulin signaling concurred with an enrichment of MET-IR complexes in *Alb-R26^Met^* hepatocytes, both in basal conditions and after insulin stimulation. This raises the possibility that in *Alb-R26^Met^* mice, increased MET levels facilitate a MET-IR complex formation that might be partially signaling compromised, at least in response to insulin stimulation, resulting in insulin resistance. Such context could lead to two distinct configurations. First, increased MET levels in *Alb-R26^Met^* hepatocytes could sequester IR, thereby preventing insulin-dependent kinase activation and signaling. This would mimic a dominant–negative effect of enhanced MET on IR, compatible to the phenotype observed in AlbDN-Met transgenics [20]. A second configuration could be that enhanced MET levels biases the activation of MET downstream signaling at the expense of IR signaling, either through competition or negative feedback mechanisms. This possibility would be coherent with basal ERK1/2 phosphorylation in unstimulated *Alb-R26^Met^* hepatocytes (predominantly downstream of MET), whereas AKT phosphorylation relayed on either HGF or insulin treatment. Additionally, this would also be consistent with synergistic effects of insulin and HGF-mediated glucose uptake stimulation, only observed when, for example, a minimal dose of insulin is used [20]. Future studies using combinatorial gain and loss-of-function approaches will further clarify reciprocal crosstalk between HGF/MET and the critical nodes of IR-mediated signaling. When searching public databases, a positive correlation between *MET* and *INSR* levels was found in obese patients with T2D either in men and women, thus possibly supporting their relationship. In this regard, although our studies have been conducted only in male mice, further studies are necessary to address possible similarities and specificities linked to gender and those related to insulin sensitivity.

Outcomes from studies mentioned herein may also shed light on putative mechanisms linked to tumor initiation as a consequence of tissue homeostasis destabilization. Indeed, the impairment of glucose homeostasis we found in male *Alb-R26^Met^* mice aggravates with aging, which notably manifested at 10 months of age. This is coincident with the time when spontaneous liver tumors start to be found in *Alb-R26^Met^* mice (40 weeks of age), with a tumor frequency reaching about 80% in older mice [12]. It is tempting to speculate that alterations in glucose homeostasis, occurring before the appearance of liver tumors, could be among key events at the root of liver tumorigenesis in *Alb-R26^Met^* mice. Such configurations could be particularly relevant for clinical prediction of patients with glucose metabolic dysfunction and insulin resistance and for therapeutic intervention, particularly for HCC associated with enhanced MET expression/activation.

## Figures and Tables

**Table 1:** List of primary antibodies. Antibodies were diluted in TTBS supplemented with 0.4% BSA at the indicated dilutions.

Antibody	Commercial Brand	Reference	Dilution
Rabbit mAb phospho-AKT Ser473	Cell Signaling Technologies	4058	1:2000
Rabbit mAb phospho-AKT Thr308	Cell Signaling Technologies	4056	1:1000
Rabbit mAb AKT	Cell Signaling Technologies	4691	1:20,000
Rabbit mAb phospho-IRβ	Cell Signaling Technologies	3024	1:1000
Rabbit mAb IRβ	Cell Signaling Technologies	3025	1:2000
Rabbit mAb phospho-ERK1/2	Cell Signaling Technologies	4370	1:4000
Rabbit pAb ERK1/2	Cell Signaling Technologies	9102	1:4000
Mouse mAb MET	Santa Cruz Biotechnologies	sc-8057	1:1000
Mouse mAb MET	Cell Signaling Technologies	3127	1:1000
Mouse mAb α-Tubulin	Sigma-Aldrich/Merck	T5168	1:5000
Mouse mAb Vinculin	Santa Cruz Biotechnologies	sc-73614	1:7500

## Data Availability

Not applicable.

## Supplementary Materials

The following supporting information can be downloaded at: https://www.mdpi.com/article/10.3390/cells11050793/s1, Figure S1: Young Alb-R26Met mice show a trend of pyruvate intolerance.
