# Supplementary material for: Enhanced Wild-Type MET Receptor Levels in Mouse Hepatocytes Attenuates Insulin-Mediated Signaling
Source: Cells. 2022 Feb 24;11(5):793. doi: 10.3390/cells11050793 (PMC8909847; doi:10.3390/cells11050793)
Supplement: Supplementary file 1 [file cells-11-00793-s001.zip › SUPPLEMENTARY FIGURES.pdf]

Supplementary Figure

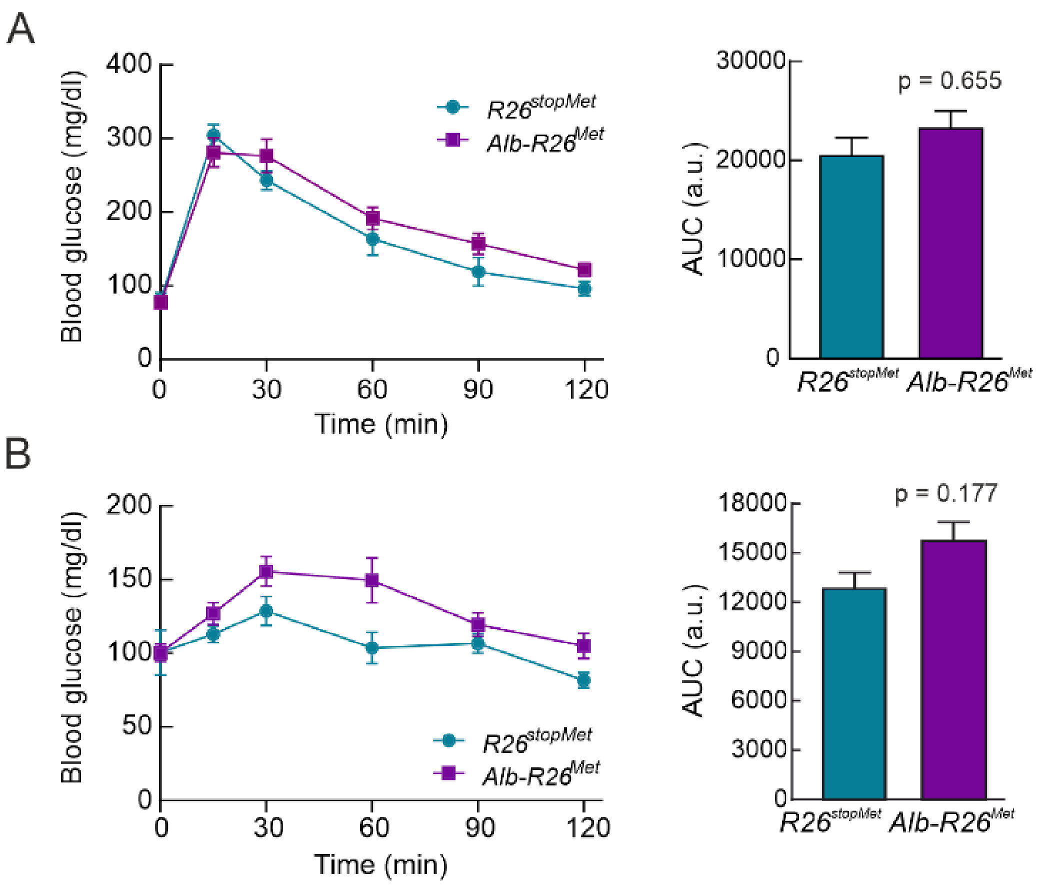

Figure S1. Young Alb-R26^{Met} mice show a trend of pyruvate intolerance. (**A**) GTT in 6-month-old *R26^{stopMet}* and *Alb-R26^{Met}* mice (*n* = 5 and 19 mice per group, respectively). Graph depicts the area under the curve (AUC) from GTT. (**B**) PTT in *R26^{stopMet}* and *Alb-R26^{Met}* mice (*n* = 5 and 13 mice per group, respectively). Graph depicts the AUC from PTT. (**A**,**B**) Values correspond to mean ± SEM.
